# Rapid desensitization for brentuximab vedotin (Adceteris^®^) allergy: a case report

**DOI:** 10.1186/s12948-018-0100-0

**Published:** 2018-10-29

**Authors:** Attilio Di Girolamo, Marcello Albanesi, Alessandro Sinisi, Eustachio Nettis, Danilo Di Bona, Maria Filomena Caiaffa, Luigi Macchia

**Affiliations:** 1 Department of Emergency and Organ Transplantation, School and Chair of Allergology and Clinical Immunology, University of Bari-Aldo Moro, Piazza Giulio Cesare, Policlinico, 70124 Bari, Italy; 2 Department of Medical and Surgical Sciences, School and Chair of Allergology and Clinical Immunology, University of Foggia, Via Luigi Pinto 1, 70100 Foggia, Italy

**Keywords:** Anti CD30, Auristatin-E, Hodgkin lymphoma, Desensitization, Monoclonal antibodies, Drug allergy

## Abstract

**Background:**

Brentuximab vedotin (BV) is an antibody–drug conjugate formed by an anti-CD30 chimeric IgG_1_ conjugated with monomethyl-auristatin-E. BV targets the CD30^+^ cells, which characterize Hodgkin lymphoma as well as anaplastic large cell lymphoma. Once bound to the CD30^+^ cells BV exerts its cytotoxic effect via the monomethyl-auristatin-E moiety. So far, accounts on immediate adverse reactions to BV remain anecdotal. Moreover, few reports exist on desensitization for BV.

**Case presentation:**

A 20-year old male patient was diagnosed with Hodgkin lymphoma in July 2014. The first line treatment with adriblastine, bleomicine, vinblastine and dacarbazine lead to a partial remission. Thus, a treatment with BV was started. However, during the second BV infusion, he developed generalized urticaria and dyspnea. In order not to discontinue the treatment with BV, we performed a thorough allergological workup and designed a 12-step rapid desensitization protocol. Overall the desensitization procedure was well tolerated and no major adverse reactions occurred.

**Conclusion:**

Rapid desensitization is a suitable and safe option in the case of BV allergy and prevents the BV treatment withdrawal.

## Background

Brentuximab vedotin (BV) is an antibody–drug conjugate formed by an anti-CD30 chimeric IgG_1_ conjugated with the anti-microtubule agent monomethyl-auristatin-E. BV represents a valid option for patients suffering from relapsing Hodgkin lymphoma and anaplastic large cell lymphoma. Indeed, BV targets CD30^+^ cells, which characterize these hematologic conditions, and exerts a potent cytotoxic effect via the monomethyl-auristatin-E moiety [1].

So far, accounts on immediate adverse reactions to BV remain anecdotal. Moreover, few reports exist on desensitization approaches with BV [2–5].

Since the introduction of monoclonal antibodies (mAbs) in therapy, adverse reactions, including hypersensitivity reactions (HSRs), have been described. In these cases, usually the diagnostic process includes skin testing (skin prick test and intradermal tests) with the offending agent [6].

Skin prick tests are performed with full-strength solution of the offending agent. As for the intradermal tests, 1:10 and 1:100 dilutions (obtained from the full strength solution) are commonly used on empirical basis. However, according to the literature, the sensibility of the skin tests in mAb allergy remains to be assessed [7].

In patients with a history suggestive of HSRs to mAbs, rapid desensitization protocols have been described and proved effective [7]. This desensitization approach is based on intravenous infusion of the offending mAb at increasing doses. Rapid desensitization is achieved by 12 consecutive steps (usually; using 3 increasing mAb concentrations). At each step the rate of drug administration is increased by 2- to 2.5-fold. The time between the different steps is 15 min.

Hereby we describe a case of a 20-year old man with Hodgkin lymphoma that developed HSR to BV and was successfully treated with a rapid desensitization protocol, adapted from Castells [7].

## Case presentation

A 20 year old patient was diagnosed with Hodgkin lymphoma in July 2014. Thus, the patient was treated with 6 cycles of adriblastine, bleomicine, vinblastine and dacarbazine. This therapeutic approach was well tolerated and initially lead to a partial remission. However, the patient experienced a relapse. Upon a second line attempt and a further relapse, the patient started BV (1.8 mg/kg) every 3 weeks. The first administration was tolerated without side effects. However, during the second infusion, he developed generalized urticaria and dyspnea. The infusion was halted and hydrocortisone (500 mg) and chlorpheniramine (10 mg) were administered with resolution of symptoms. No epinephrine was required. The patient was then referred to our clinic.

Considering the immediate nature of the reaction and the rapid response to anti-allergic treatment, a thorough allergological workup was performed with the purpose of desensitizing the patient, in consideration of the need for avoiding discontinuation of BV, as recommended by the Haematologists.

Thus, we performed skin prick tests and intradermal tests. For the skin prick tests, we used BV full-strength solution (5 mg/ml). For the intradermal tests, we used increasing concentrations of BV (viz 0.00044, 0.0044, 0.044 mg/ml, respectively). Histamine (10 mg/ml) and saline were used as the positive and the negative control, respectively. Both skin tests and intradermal tests proved negative, for all the concentrations used.

In spite of these results, but considering the necessity of treatment maintenance, we devised and implemented a 3-bag 12-step protocol of rapid desensitization. Pre-medication included omeprazole (40 mg), chlorphenamine (10 mg), ondansetron (5 mg) and dexamethasone (4 mg). Thus, we used 3 BV dilutions at increasing concentration: 0.0044, 0.044, 0.44 mg/ml. The target dose was 180 mg, intravenously (calculated on patient body weight). The desensitization protocol is reported in Table 1.

**Table 1:** BV desensitization protocol

Step	Solution (mg/ml)	Step time (min)	Infusion rate (ml/h)	Drops/min	Total drops	Volume (ml)^a^	Dose (mg)
1	0.0044	15	4	3/2	20	1	0.0044
2	0.0044	15	10	4	60	3	0.0132
3	0.0044	15	20	6	100	5	0.022
4	0.0044	15	40	14	200	10	0.044
5	0.044	15	10	4	60	3	0.132
6	0.044	15	20	6	100	5	0.22
7	0.044	15	40	14	200	10	0.44
8	0.044	15	80	26	400	20	0.88
9	0.44	15	20	6	100	5	2.2
10	0.44	15	40	14	200	10	4.4
11	0.44	15	80	26	400	20	8.8
12	0.44	154	150	50	8000	386	169.85

^a^1 ml = 20 drops

Overall the desensitization procedure was well tolerated and no major adverse reactions occurred. Using this desensitization scheme, three administrations of BV were delivered at target dosage. Only two urticarial wheals occurred during the final step of the first desensitization. However, we continued the infusion and completed the first administration. During the following desensitization procedures the patient lamented only transient pruritus without wheals.

## Discussion and conclusion

Rapid desensitization was proven to be effective in HSRs due to several chemioterapic such as cytarabine, carboplatin, rituximab. In line with previous accounts [2–5], our report demonstrates that rapid desensitization for BV is effective and safe. The desensitization protocol described in this report required 319 min (an suitable time compared to other protocols; Table 2).

**Table 2:** Comparative table of BV desensitization protocols

Protocol	Total steps	Total elapsed time (min)	Step time (min)	Successful	Procedure discontinuation
Fizesan [2]	17	405	15^a^	Yes	No
O’Connell [3]	12	323	15	Yes	No
De Vita [4]	13	155	10; 15^b^	Yes	Yes^c^
Arora [5]	12	365	15	Yes	No
Di Girolamo/Albanesi	12	319	15	Yes	No

All single cases

^a^Steps from 1 to 15 → 15 min each; step 16 → 30 min

^b^Step from 1 to 8 → 10 min; step from 9 to 13 → 15 min

^c^Infusion suspended and then resumed

Importantly, in almost all the desensitization protocols described for BV, the time between each step is at least 15 min, as in our case. This interval time seems to be pivotal in desensitization procedure to avoid major adverse reactions that might lead to the desensitization withdrawal (Table 2). Indeed, in vitro models of basophil desensitization demonstrated that human basophil from allergic patients, when repeatedly incubated with suboptimal doses of the allergen, reached a maximal unresponsiveness when the incubation time was between 15 and 30 min [8].

In the case presented skin tests were negative, as for other reports [2–5], nonetheless we carried out the desensitization in consideration of the clinical features of the case, typical of HSR. According to a previous report from O’Connel et al. [3], we believe that the predictive value of skin testing for BV allergy remains ambiguous. In fact, even though the diagnostic tests proved negative, our patient had urticaria and pruritus during the first desensitization, suggesting that the adverse reaction was allergological in nature.

Indeed, the negativity of the skin tests might be due to different reasons: (i) the BV concentrations used for the skin tests were determined on empirical basis, therefore the allergen amount might had been not sufficient to elicit a valid response; (ii) HSRs to BV (as for other mAbs) might be due to other immune mechanisms not necessarily involving IgE (e.g. complement activation).

Moreover, during the first desensitization procedure the patient showed mild urticarial lesions but not during the following two desensitization procedures. This observation suggests that after rapid desensitization a certain degree of tolerance might be induced.

In conclusion: (i) the desensitization protocol proposed proved to be amenable; (ii) desensitization is an effective and safe option in the case of BV allergy; (iii) each step in the rapid desensitization procedure should be at least 15 min; (iv) standardization in a larger cohort of patients would be required to enhance the diagnostic power of skin tests in BV allergy.

## Abbreviations

BV: brentuximab vedotin
mAbs: monoclonal antibodies
HSRs: hypersensitivity reactions
